# “We Don’t Do Any of These Things Because We are a Death-Denying Culture”: Sociocultural Perspectives of Black and Latinx Cancer Caregivers

**DOI:** 10.21203/rs.3.rs-3470325/v1

**Published:** 2023-10-26

**Authors:** CANDIDUS C NWAKASI, Darlingtina Esiaka, Chizobam Nweke, Kate de Medeiros, Wilson Villamar, Runcie Chidebe

**Affiliations:** University of Connecticut; University of Kentucky; University of Connecticut; Concordia University; Roger Williams Medical Center; Miami University

**Keywords:** Latinx cancer survivors, cancer caregiving, care stewardship, cancer health equity, health care access, culture and health

## Abstract

This qualitative study explored the unique challenges and experiences faced by Black and Latinx cancer survivors through the lens of their caregivers, including the specific cultural, social, and systemic factors that influence cancer survivorship experience within these communities in the United States. We conducted six focus group discussions (three Latinx and three Black groups) with a total of 33 caregivers of cancer survivors, (Mean age = 63 years). Data were analyzed using inductive content analysis; The sociocultural stress and coping model was used as a framework to interpret the findings. We identified three main themes: 1) families as (un)stressors in survivorship such as the vitality of social connections and families as unintended burden; 2) responses after diagnosis specifically whether to conceal or accept a diagnosis, and 3) experiencing health care barriers including communication gaps, biased prioritizing of care, and issues of power, trust, and need for stewardship. This study’s findings align with previous research, highlighting the complex interplay between cultural, familial, and healthcare factors in cancer survivorship experiences within underserved communities. The study reiterates the need for culturally tailored emotional, physical, financial, and informational support for survivors and their caregivers. Also, the study highlights a need to strengthen mental health and coping strategies, to help address psychological distress and improve resilience among survivors and their caregivers.

## Introduction

The presence of social support and the involvement of family caregivers in the care of a cancer patient are essential contributors to cancer survivorship experiences [1, 2]. In Black and Latinx communities, where patients often encounter race-based barriers when accessing diagnosis and treatment, cancer caregivers provide emotional, physical, and practical support systems that improve the survivorship experiences of cancer survivors [3–5]. The caregiving structure in Black and Latinx communities includes aspects such as family dynamics, community support, and access to health care services [6]. Also, it leverages the cultural backgrounds and traditions that influence perceptions of illness and caregiving [7]. For example, religious and spiritual beliefs may play a significant role in cancer care and support and are more likely to be incorporated into caregiving experiences by Latinx and Black caregivers of racialized cancer patients [8, 9], thus influencing the well-being and survivorship experiences of cancer care receivers.

Cancer survivorship experiences and adequate caregiving in Black and Latinx communities are often marred by challenges, including limited access to health care, financial constraints, and disparities in health care quality [10, 11]. While cancer survivors navigate cancer diagnosis, associated health and lifestyle changes, and the implication of diagnosis on their relationship with families and communities, their caregivers may struggle with accepting the health condition of their loved one, balancing caregiving responsibilities and work, and may lack the resources to provide optimal care [12–14]. These experiences of survivors and their caregivers can be intricately interwoven, resulting in a significant effect on the cancer survivorship experience [12, 13]. Given the role of caregivers in the wellbeing of cancer survivors in minoritized populations, more understanding of cancer survivorship through the lens of caregivers in Black and Latinx communities is crucial. By focusing on the perspectives of caregivers who support Black and Latinx cancer survivors, the study seeks to gain insights into specific cultural, social, and systemic factors that influence the cancer survivorship experience within these communities.

## Method

We used a qualitative descriptive design for our inquiry. This approach is often used in healthcare research when there is not a deep theoretical aim but there is a need to stay close to the subjective views of the participants [15].

### Setting and participant recruitment

The PI’s (lead author) Institutional Review Board approved this study. The study participants (referred to throughout as caregivers) were family members and friends of cancer survivors in the New England region of the U.S. The inclusion criteria were: 18 years or older, self-identify as Black or Latinx or both, identify as a person with a history of caring for a family member or friend with cancer, and able to complete the interview in English or Spanish. Each participant provided written informed consent and was given a $50 cash gift for their time. We used multiple approaches to recruit the participants. First, we engaged two trusted community leaders, a Latinx chaplain and a Black reverend, who helped lead the community recruitment teams. Second, research team members engaged with communities affected by cancer which helped raise awareness about our research and expanded our understanding of issues affecting cancer survivors. Third, during the individual interviews involving cancer survivors, we used a snowball method of sample recruitment whereby cancer survivors shared the study with their caregivers and others in their community to gain their interest in participating.

### Procedure and data collection

The authors have a combined 40-plus years of experience conducting qualitative studies. For the current study, we conducted six in-person focus group discussions (FGD) between January and March 2023. Three focus groups included Black caregivers of Black people with a history of cancer. The other three focus groups included Latinx caregivers of Latinx individuals with a history of cancer. Four FGDs were conducted in English, and two in Spanish. Participants provided information about their age, gender, education, relationship status, race and ethnicity, and type of relationship with a cancer survivor (e.g., friend or family). The FGDs ranged from 56 to 96 minutes; Each FGD was audio recorded.

The three FGDs with Black participants were conducted in an African American church in English, and moderated by the first author. The first author also moderated the FGD session with Latinx participants, which was conducted in English. Although the first author was present during the two Spanish FGDs involving Latinx cancer caregivers, a Spanish-speaking research team member moderated the sessions. The FGD participants responded to open-ended, semi-structured questions that explored cancer survivorship experiences (See Table 1 for questions).

### Data analysis

All six audio recordings of the FGDs were professionally transcribed. The two Spanish transcripts were translated into English by a bilingual member of the research team before analysis. We used Dedoose (version 9.0.46) for data management. Inductive content analysis was used to identify themes relevant to the research aims. Content analysis involves a systematic and objective analysis of texts, over multiple stages, to make meaningful inferences about the studied phenomenon [16, 17]. FGD texts were carefully read and re-read to identify meaningful units (e.g., a sentence or a short paragraph) to develop codes. Next, coded data were extracted, and evaluated for their appropriateness by two research team members. The codebook was refined as well. Afterward, the excerpted data were extracted by code, reviewed, and grouped into concept categories which we refer to as sub-themes. The sub-themes were reviewed and nested within larger themes to address the study aims.

### Findings

The focus groups included 33 participants, 24 women and 9 men. Their age ranged from 35 to 86 years. Although some participants provided information about cancer types, the study’s focus is not on cancer types (see Table 2 for more participant information). We identified three main themes: families as (un)stressors in survivorship, responses after diagnosis, and experiencing health care barriers. Each theme contains sub-themes, which are presented below including illustrative quotes.

### Families as (un)stressors in survivorship

Participants’ responses indicated that families and friends are essential components of the cancer survivorship experience — adding to the stress or helping to relieve the stress from cancer. The sub-themes include the vitality of social/familial connections, and families as unintended burdens and stressors.

The vitality of social/familial connections. Participants described how important a strong family connection is for a person with cancer — cancer survivors rely on them for care and are sometimes confident that care and support will be there when needed. One of the participants in Group 5 said: “I think in the Latino community, we always have more family support.” Supports, particularly psychological and instrumental supports, are important for cancer survivors and bring them positive affect. These supports may come from estranged family members who readily show up to care for a cancer survivor. A participant from Group 2 explained:

We weren’t married anymore, but I went to hospice almost every single day. And I’d just sit. His sisters were there and everything and we would [stay] we had a birthday party for my son there. Even though he couldn’t eat anything, he asked for cheesecake, but he couldn’t eat it. But we tried to keep his spirits lifted and everything.

Families as unintended burdens and stressors. Some participants’ descriptions implied that while the bond between relatives can benefit cancer survivors, sometimes, families and friends may be the stressors that result in a negative survivorship experience. This may be from family expectations about what the cancer survivor should or should not do for their recovery and health. For example, a participant from Group 4 described:

If I’m [the cancer survivor is] not doing what they want me [them] to do, then they’re going to treat me a certain way, but if I do what they want me to do, it doesn’t feel right for me. And it’s almost like you come into this catch-22 place.

Another participant from Group 5 mentioned how the actions of family members, even when borne out of goodwill, can affect the sense of autonomy of cancer survivors. The participant said: “They [cancer survivors] want to do something…oh, no, no, you can’t do that. I’m going to do that for you. So, we take [control] away from them.” Caring for a person with cancer can be stressful. When caregivers are stressed, tensions related to meeting the expected caregiving easily arise between caregivers and cancer survivors. Thus, resulting in a more stressful situation for the family including the cancer survivors. A participant from Group 2 said: “I think that they [families] are stressed, and they don’t know how to release that, so they attack each other.”

### Responses after diagnosis

A sense of interdependence affects how underserved communities understand or respond after a cancer diagnosis. The sub-themes to describe how families from underserved communities navigate cancer within the confines of interdependence are: concealing diagnosis and embracing diagnosis.

Concealing diagnosis. According to some participants, a cancer diagnosis is private and others may see survivors as weak or less than when they know about their diagnosis. A participant in Group 4 explained: “Sometimes it’s a matter of fear, just telling too many people, and people looking at you or treating you as if you’re sick.” Some cancer survivors do not share their diagnosis with their family members because they do not want their loved ones to experience the cancer burden with them. A participant in Group 2 said: “My second husband, he had it and we didn’t know.” The participant went further to add that they learned about his condition only when he was transferred to hospice, and they went to see him:

He [my son] started screaming and yelling at me and saying, Mom, why’d you bring me here knowing that my father was dying? I said I didn’t know. I did not know until we got to the hospital with the people from the hospice to find out that he was dying. It was really, really bad. He’s from Nigeria.

Concealing diagnosis may limit the support a family battling cancer may get in their community as a participant from Group 3 explained: “…that’s a negative for our community. I mean, there are things you don’t want to share, but cancer?”

Embracing diagnosis. Beyond cancer diagnosis and prognosis, sometimes, family members struggle to make sense of the full extent of a cancer-associated burden. Like the sub-theme about concealing diagnosis, some participants mentioned that family members whom cancer survivors depend on for care might be in denial of the diagnosis. This fractures the support for cancer survivors in the family. A participant in Group 5 described why relatives do not visit a person with cancer who needs help:

So, it is a form of denial as well. It’s not happening, I don’t see it, it’s not happening. And the other thing is that it’s also a form of not getting involved, not knowing that the person is there or that the person is not suffering.

Some participants added that cancer denial can be intense and that families do not want to come to terms with the person dying. Thus, the cancer survivor may not get the psychological support needed to resolve their affairs. Group 1 participant explained: “…we Latinos, we don’t do power of attorney…we don’t do advance directives. We don’t do Five Wishes. We don’t do any of these things because we are a death-denying culture.”

### Experiencing health care barriers

Participants described some barriers Black and Latinx families with cancer face in healthcare facilities. The sub-themes include communication gaps, prioritized care based on race/ethnicity, and power, trust, and stewardship.

Communication gaps. The experience of communication gaps between cancer survivors and health care providers which include conflicting medical instructions resonated among the focus groups. Latinx participants identified that the language barrier between cancer survivors and health care providers was a communication gap which highlights the importance of having a caregiver or a trusted relative as a translator/interpreter. Further, the presence of communication barriers influences outcomes for cancer survivors including their ability to follow medication regimens, describe symptoms to health care providers, and understand critical health care decisions. As stated by a participant in Group 5:

One [a provider] gives you one piece of information, the other [another provider] gives you another piece of information, the next one [a third provider] gives you another piece of information. They confuse you with the information they give you. Then, you don’t know where to go, because in reality, you don’t know about medicine.

Prioritized care based on race/ethnicity. Participants identified healthcare barriers that made cancer survivorship difficult in their communities — some health care providers have racial and ethnic biases. A Group 1 participant commented: “Sometimes they give more priority to other races or other cultures than the African Americans or the Africans and the Latinos.” Some participants mentioned that taking care of families with cancer made them more attentive to cancer health including routine cancer screening at their primary care providers. However, racial and ethnic bias during their visits reminded them that they are not prioritized during health care encounters. A participant offered more insight into racial bias in health care for cancer survivors by recalling their experience with a physician. The participant said: “I have a primary care doctor. And he said to me, *you’ve been neutered, right*?” She described further with a pained tone in her voice:

I’ll never forget him. And I said, animals get neutered. You’re calling me a neutered? Are you calling me an animal? And he was like, Oh, no. Don’t… I said, excuse me?! I got hot, hopped off that table, took that gown off. He was looking at me like, where are you going? I’m out of here.

Power, trust, and stewardship. Cancer survivors’ caregivers (families and friends) know that racially and ethnically biased health care exists. They intentionally try to navigate these situations in healthcare institutions where they see themselves as disadvantaged due to not having enough power, and at the same time not trusting institutions that have historically been involved in oppressing them. Thus, they become care stewards for their loved ones with cancer in a health care setting. A Group 2 participant explained: “We always leave someone [a relative] there for help, assistance. You always try to have someone.” This may also protect them from harm as a Group 3 member described: “I want to know everything and hear everything and see the way he’s being treated.” Stewardship may be about helping cancer survivors ask important questions they ordinarily would not ask because they do not trust their medical providers. A group 1 participant mentioned: “So, there is a lot of questions that people don’t ask. They embark in the system, and because they don’t trust, they don’t communicate.”

## Discussion

This study explored cancer survivorship experiences from the perspective of caregivers in underserved communities. These experiences are summarized under three main themes: families as (un)stressors, responses following diagnosis, and healthcare barrier experiences. We found that a family’s culture of offering support plays a central role in cancer survivorship experiences because they lead to the formation and maintenance of social connectedness. These caregiving support present mainly in the form of emotional, physical, and psychological support [10, 18]. While caregiving may help relieve stress for cancer survivors, we also found that families including caregivers may also contribute to stressful cancer survivorship experiences. This may result from caregivers restricting a cancer survivor’s autonomy or having unrealistic expectations from them. Additionally, because caregiving may be perceived as obligatory [19], cancer survivors may receive poor quality care or be exposed to negative emotional situations. Our findings agree with studies by [20] and [21].

In our study in Black and Latinx communities, responses to cancer diagnosis such as concealing or denying the presence of cancer vary and arise from personal and cultural factors. While some individuals withhold cancer diagnosis as a coping strategy because they worry about being perceived as sick/weak, others do so to prevent their families from experiencing the emotional burden of cancer. Such unwillingness to discuss cancer diagnosis was documented by [22] and was found to be closely related to stigmas associated with certain types of cancers. Furthermore, certain cultures struggle with acknowledging death, and within such cultures, it is not unusual to have relatives deny a cancer diagnosis even though the affected individual has accepted their fate. This may be because acknowledging the disease means accepting the nearness of death or the likelihood of dying [23]. This death-denying attitude may explain why some Black and Latinx people would rather focus on ineffective treatments instead of utilizing end-of-life care as reported in several studies [23–25].

Black and Latinx cancer survivors and their families in our study received conflicting instructions and information from different health care providers, causing confusion. Language barriers and the absence of clarification by healthcare professionals also contributed to the widening of an already-existing communication gap. Similar communication problems resulting from language barriers have been reported in the past [26–28]. Although there is a desire for trust between health care providers and families of cancer survivors, the perception that information on cancers is lacking and poor communication, may limit trusting provider-patient relationships in these communities.

There is a predominant feeling of exposure to ethnic/racial bias among participants, who believe this to be responsible for poor cancer care, lack of health information, and use of unprofessional language/conduct — all experienced by minoritized people in healthcare settings. Some studies have also reported similar racial disparities in cancer diagnosis and care [29, 30]. With regards to cancer diagnosis, [22] reported mistrust of the healthcare system among Black and Latinx populations in the U.S. Due to mistrust, informal caregivers in our study try to become much more involved in the survivors’ primary care. They act as stewards for their families in healthcare settings to protect their loved ones from poor care quality, and mistreatment.

### Limitations

The current study has some limitations. The experiences of Black and Latino families in New England may not be generalizable to that of other families in the U.S. and beyond. The interviews conducted in Spanish were transcribed and translated into English prior to data analysis. It is possible that a loss of nuance and meaning occurred. Also, Spanish concepts may have been loosely translated due to a lack of English equivalence. Many of the participants in the current study are from similar educational and socioeconomic backgrounds. As such, their experience may not apply to others of diverse backgrounds. Finally, while FGD can provide valuable insights, the group dynamics may have impacted participant responses or resulted in social desirability bias. The FDG participants were selected based on specific criteria, and their views might not represent the perspective of their larger communities.

## Conclusion

This study delves into the experiences of cancer survivorship through the lens of informal caregivers in underserved communities. Three main themes emerged from these experiences: the impact of families as sources of stress or support, varied responses to cancer diagnosis rooted in personal and cultural factors, and challenges in healthcare access and communication. Family support, encompassing emotional, physical, and psychological aid, is pivotal in survivors’ well-being. However, caregivers can inadvertently contribute to stress by limiting autonomy or having unrealistic expectations. This study’s findings align with previous research, highlighting the complex interplay between cultural, familial, and healthcare factors in cancer survivorship experiences within underserved communities.

## Figures and Tables

**Table 1:** 

Questions in the Study Interview Guide
What, in your opinion, are some of the challenges of cancer survivorship?
What are the contributions of family and friends in helping people in the community with cancer?
Some people say cancer is stigmatizing to the person and family with cancer. What do you think about this and why?
What are some examples or acts of health care providers towards your relative with cancer that made you feel good or bad about your experience?

**Table 2:** Demographic Characteristics of Participating Caregivers of Cancer Survivors

Number of Participants	Age (Years)	Education	Race/Ethnicity	Relationship Status
33 Participants 24 Women 9 Men	Range: 35–86 Mean: 63	20 Attended college or higher 13 Attended Highschool	15 Latinx 18 Black	12 Married 13 Single 4 Divorced 4 Widow
